# Female Asthmatic Patients Have Higher Risk to Develop Gemifloxacin-Associated Skin Rash, Highlighting Unique Delayed Onset Characteristics

**DOI:** 10.3390/antibiotics8030134

**Published:** 2019-08-31

**Authors:** Chiou-Mei Wu, Po-Ju Wei, Yu-Ting Shen, Hsu-Liang Chang, Ying-Ming Tsai, Hung-Fang Pan, Yong-Chieh Chang, Yu-Ching Wei, Chih-Jen Yang

**Affiliations:** 1 Department of pharmacy, Kaohsiung Municipal Ta-Tung Hospital, Kaohsiung Medical University Hospital, Kaohsiung Medical University, Kaohsiung 80145, Taiwan; 2 Department of Internal Medicine, Kaohsiung Municipal Ta-Tung Hospital, Kaohsiung Medical University Hospital, Kaohsiung Medical University, Kaohsiung 80145, Taiwan; 3 Division of Pulmonary and Critical Care Medicine, Department of Internal Medicine, Kaohsiung Medical University Hospital, Kaohsiung Medical University, Kaohsiung 80145, Taiwan; 4 Department of pathology, Kaohsiung Municipal Ta-Tung Hospital, Kaohsiung Medical University Hospital, Kaohsiung Medical University, Kaohsiung 80708, Taiwan; 5 School of Medicine, College of Medicine, Kaohsiung Medical University, Kaohsiung 80145, Taiwan

**Keywords:** Gemifloxacin, adverse drug reaction, skin rash, fluoroquinolones

## Abstract

Gemifloxacin is a common oral antibiotic for lower respiratory tract infection worldwide. We noticed an uncommon delayed onset skin rash in patients who received Gemifloxacin. Therefore, we retrospectively reviewed all patients who received Gemifloxacin from 1 January 2011 to 31 May 2016 in a university-affiliated hospital in Taiwan. A total of 1358 patients were enrolled, of whom 36 (2.65%) had skin eruptions. The female patients had a significantly higher odds ratio (OR) 2.24 (95% confidence interval (CI) 1.11–4.53, *p* = 0.021) of having skin eruptions. A history of asthma was also a significant risk factor (OR 2.04, 95% CI = 1.01–4.14, *p* = 0.043). Female asthmatic patients had the highest risk of skin eruptions (10/129, 7.2%) with an adjusted OR up to 4.45 (95% CI = 1.81–10.93, *p* < 0.001) compared to male and non-asthmatic patients. Of note, up to 58.3% (21/36) of the patients experienced a skin rash after they had completed and stopped Gemifloxacin. The median onset time was on the second day (ranging one to five days) after completing treatment. We reported that female asthmatic patients have the highest risk of Gemifloxacin-associated skin eruptions in Asia and that they highlighted a unique delayed onset skin rash.

## 1. Introduction

Fluoroquinolones are considered to be safe, well-tolerated antibiotics and are commonly used to treat lower respiratory tract infections [1,2,3,4]. Gemifloxacin is a relatively new and broad-spectrum oral fluoroquinolone antibiotic. It has the ability to inhibit both DNA gyrase and DNA topoisomerase IV enzyme systems [5,6,7]. Gemifloxacin has an oral bioavailability of approximately 71%. Approximately 20–35% of Gemifloxacin is excreted unchanged in the urine after 24 h. The elimination half-life of Gemifloxacin is 6–8 h in patients with normal renal function, supporting once daily dosing [1].

Gemifloxacin has been shown to be potent against multi-drug resistant pneumococcus [8,9]. Thus, it has become increasingly popular to treat community acquired pneumonia worldwide [3,5,6,10,11,12]. In addition, Gemifloxacin exerts less activity against mycobacterial tuberculosis compared with other respiratory fluoroquinolone. Therefore, it has been proposed that the use of Gemifloxacin instead of other fluoroquinolones can prevent mycobacterial tuberculosis treatment delay while preserving the advantages of fluoroquinolones for community-acquired pneumonia treatment [13].

Several large-scale studies showed that the Gemifloxacin-related adverse events included diarrhea, rash, and nausea. Of note, Gemifloxacin had higher incidence of skin rash when compared with other fluoroquinolone antibiotics for the treatment of lower respiratory tract infection [2,3,14,15,16].

The proposed risk factors for Gemifloxacin-associated rash in Western countries include female gender, an age younger than 40 years, and postmenopausal women receiving hormone replacement therapy, but almost no reports focused in Asia exist [1,2,10,15,17]. A recent prospective study in India revealed that Gemifloxacin-induced skin rash was detected in one patient out of two prescriptions in septicemia patients [18].

We recently recorded several patients who had delayed skin eruptions after completing Gemifloxacin therapy through our adverse drug reaction reporting system, some of whom visited our emergency room and were admitted to the hospital. Of particular note, because the patients were asked to return for a checkup on the final day of drug prescription, most of the physicians were unaware that a skin rash had occurred. The delayed skin rash was unexpected and had rarely been described in the literature.

Therefore, the aim of this study was to investigate the clinical presentations and the risk factors of Gemifloxacin-related skin eruptions through a retrospective review of all patients who received Gemifloxacin for lower respiratory tract infections at a university-affiliated teaching hospital in Taiwan.

## 2. Results

A total of 1358 patients who received Gemifloxacin treatment during the study period were enrolled, of whom 36 (2.65%) had skin eruptions (the case group) and the remaining 1322 (97.35%) patients did not (the control group). The demographic data of both groups are listed in Table 1. The female patients had a higher odds ratio (OR) of 2.24 (95% confidence interval (CI) = 1.11–4.53, *p* = 0.021), and a history of asthma was also a risk factor of having skin eruptions (OR 2.04, 95% CI = 1.01–4.14, *p* = 0.043). An age less than 40 years was not a significant risk factor for Gemifloxacin-induced skin eruptions. Drug exposure for longer than seven days had a trend of increasing the risk of a skin rash, but without significance (OR 2.25, 95% CI = 0.92–5.52, *p* = 0.077).

**Figure 1:**
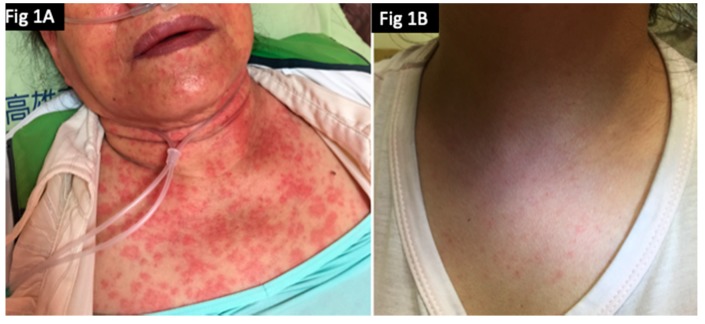
(**A**) Maculopapular rash developed two days later after completing seven-day Gemifloxacin treatment in a 76 years old female patient. (**B**) Exanthematous rash occurred on the final day of the seven-day Gemifloxacin treatment in a 32 years old female patient.

**Figure 2:**
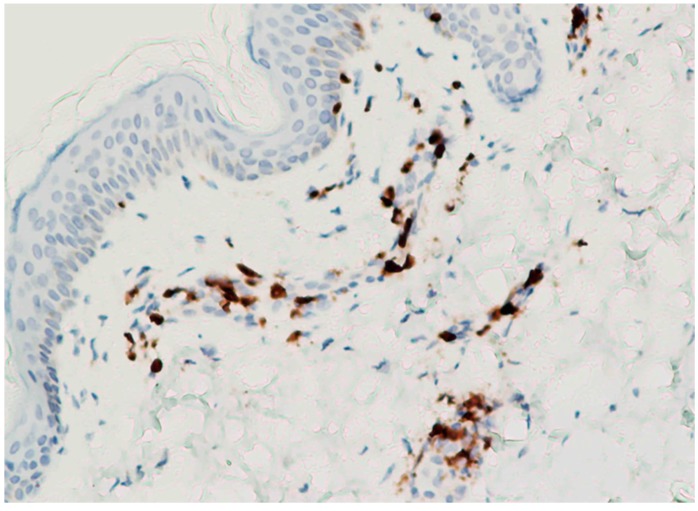
Skin biopsy in a patient who had severe skin rash. The specimen showed inflammatory infiltrates of lymphocytes and neutrophils around the vessels in the upper dermis. Some T lymphocytes, highlighted by the immunostaining of CD3, are noted at perivascular region in the dermis and at the dermo–epidermal junction (100×).

We further analyzed the risk factors using a backward selection logistic regression model to estimate adjusted odds ratios (aORs) (Table 2). The results showed that female asthmatic patients had the highest risk of skin eruptions (10/129, 7.2%; aOR 4.45, 95% CI = 1.81–10.93, *p* < 0.001). We also analyzed the clinical presentations of the patients with Gemifloxacin-related skin rashes. Thirteen of the 36 patients (13/36, 36.11%) who had a Gemifloxacin-induced skin eruption visited our emergency room, and two patients (2/36; 5.55%) were admitted to our dermatology ward, both of whom were finally discharged in a healthy condition. No risk factors for visiting the emergency room or hospitalization were identified (data not shown). In addition, 27 patients (27/34, 79.41%) had a rash with pruritus, and the remaining patients had a rash without pruritus. According to the medical charts, 14 patients (14/26, 53.85%) had a maculopapular rash and 12 patients (12/26, 46.15%) had an exanthematous rash (Figure 1A,B). A skin biopsy was performed in one of the patients who was admitted to our hospital (Figure 2), which showed inflammatory infiltrates of lymphocytes and neutrophils around the vessels in the upper dermis.

Of note, 16.6% (6/36) of the patients had skin eruptions that occurred on the final day of drug exposure, and 58.3% (21/36) of the patients had a skin rash after they had completed and stopped Gemifloxacin. The median onset time was the second day after the final day of drug treatment (ranging one to five days after completing the treatment).

## 3. Discussion

This is the first study to report on Gemifloxacin-related skin eruptions in Asia. Female asthmatic patients had the highest risk of having Gemifloxacin-related skin eruptions. Of note, we disclosed that Gemifloxacin-induced skin rashes often occur days after completing Gemifloxacin treatment. This study reminds physicians and patients about the risk of Gemifloxacin-induced skin rash and a unique delayed skin rash under or after Gemifloxacin treatment.

In clinical practice, physicians often prescribe oral Gemifloxacin to patients suffering from lower respiratory tract infections and ask them to return five to seven days later for a checkup after completing treatment. However, the delayed onset of the skin rash means that the physicians may not have been aware of the skin eruption, as it would not yet have occurred at the checkup. The delay onset skin eruption is unique.

Allergy to antibiotics can be classified as immediate or non-immediate (delayed) hypersensitivity reactions, and immediate reactions are usually IgE-mediated, whereas non-immediate hypersensitivity reactions are usually non-IgE- or T cell-mediated [19]. The clinical manifestations of antibiotic allergy may be cutaneous, organ-specific, systemic, or a combination of these manifestations. The cutaneous manifestation always presents as a skin eruption with or without pruritus [15]. Severe cutaneous adverse reactions are rare but may be life threatening, such as Stevens–Johnson syndrome or toxic epidermal necrolysis. Allergy to fluoroquinolone antibiotics is relatively less common and also includes immediate and non-immediate reactions, of which non-immediate hypersensitivity reactions are less frequent, with most cases being presented as case reports [17,20].

Files et al. reported a study showing that the clinical efficacy of Gemifloxacin once daily for five days is not inferior to seven days in patients with pneumonia, and even disclosed that several cases had skin rash after completing Gemifloxacin [2]. However, the incidence and pathogenesis remain unclear. Generally speaking, immediate skin allergy induced by fluoroquinolones is mediated by histamine release from canine cutaneous mast cells and H2-receptor stimulation [21]. Most hypersensitivity reactions to quinolones are immediate reactions, which are IgE-mediated. Importantly, reactions are severe for 70% of cases [22,23]. An immediate hypersensitivity reaction to Gemifloxacin has been reported [24].

Only a few studies have explored the mechanisms underlying non-immediate reactions to fluoroquinolones. Schmid et al. reported that these reactions may be associated with a T cell immune response against the quinolone, suggesting that the original compound is recognized by T cells without drug metabolism or processing [19,25]. Recent studies have shown that in these delayed reactions, drug-specific CD4 and CD8 T cells recognize the drug through T cell receptors in an major histocompatibility complex (MHC)-dependent manner [20,26,27]. Furthermore, the nature of drug-T cell interactions as well as cross-reactivity with other quinolones have been investigated through the generation and analysis (flow cytometry and proliferation assays) of quinolone-specific T cell clones [25]. These studies concluded that T cells are involved in delayed immune reactions to quinolones, such as Gemifloxacin, and that cross-reactivity among different quinolones is frequent [28]. We were unable to investigate cross-reactivity with other fluoroquinolones in the current study, since our hospital does not permit the prescription of Gemifloxacin to patients with a history of allergy to other fluoroquinolones.

Most specimens of Gemifloxacin-related skin eruptions show mild exanthematous eruptions, and biopsies always reveal a moderate perivascular T cell infiltration in the dermis [15]. We also found similar results (Figure 2). The specimen in Figure 2 showed inflammatory infiltrates of lymphocytes and neutrophils around the vessels in the upper dermis. Umair et al. demonstrated that Gemifloxacin has the potential to affect both innate and adaptive components of immune responses. Neutrophils and monocytes are the main players of the innate immune system and they are derived from myeloid progenitor cells. Increases in the count of both the neutrophils and monocytes indicate that Gemifloxacin may have a directly stimulating effect on myeloid progenitor cells. This observation may explain the result of neutrophils also being present in the skin biopsy in the current study [29].

However, whether the pathogenesis of Gemifloxacin-related allergy is different from other fluoroquinolone antibiotic allergies is still unclear, and further basic research is necessary.

Gemifloxacin-associated skin rash has been reported to occur more often in female patients, patients younger than 40 years of age, patients taking the drug for longer than seven days, and in postmenopausal women receiving hormone replacement therapy [2,10,14,30]. In this study, we found that asthma was also a risk factor for Gemifloxacin-related skin eruptions, but an age younger than 40 years and Gemifloxacin treatment for more than seven days were not significant risk factors. However, how these risk factors influence the development of skin rash is still unknown.

There are several limitations to this study. First, missing data are inevitable due to the nature of a retrospective study. Second, only one patient had the result of a biopsy, and the pathogenesis of Gemifloxacin-related allergy is still unknown. Third, we could not evaluate whether hormone replacement therapy in postmenopausal women was also a risk factor because hormone replacement therapy is not common in Taiwan and such information was not available in the enrolled patients. Fourth, data obtained from a single hospital may not be generalizable, and further large-scale prospective studies are needed to validate our findings.

In conclusion, this is the first study to report that female asthmatic patients are at the highest risk of having Gemifloxacin-induced skin eruptions. The median onset time of such eruptions was two days after completing treatment. This study should remind physicians and patients to pay attention to a very delayed skin rashes after completing Gemifloxacin treatment.

## 4. Methods

### 4.1. Study Population

We retrospectively reviewed all patients who received Gemifloxacin for lower respiratory tract infections at the Kaohsiung Medical University-affiliated Kaohsiung Municipal Ta-Tung Hospital from 1 January 2011 to 31 May 2016.

Patients who had Gemifloxacin-related skin rashes were identified via the reporting system of adverse drug reactions from the hospital. All of the enrolled patients had a skin rash that developed after Gemifloxacin therapy and excluded other possible drugs. Each case with a skin rash possibly induced by Gemifloxacin was assessed for entry into this study by the consensus of two experts, including a physician (Chih-Jen Yang) and a pharmacist (Chiou-Mei Wu). Of note, Gemifloxacin was not prescribed for any patient with a history of allergies to fluoroquinolone antibiotics at our hospital.

### 4.2. Study Outcomes and Covariates

We recorded demographic data from medical charts including age, gender, a history of allergy to antibiotics, non-steroidal anti-inflammatory drugs, other drugs such as contrast agents, and data on comorbidities such as asthma and allergic rhinitis. We also recorded the characteristics of the skin rash including the presence or absence of pruritus, as well as two distinct patterns: maculopapular rash and exanthematous rash. We documented any emergency room visits and hospitalizations due to severe skin eruptions after Gemifloxacin treatment.

### 4.3. Ethical Consideration

The Institutional Review Board of Kaohsiung Medical University Hospital approved this study (KMUHIRB-E(I)-20150166). Considering the retrospective nature of the study, the Institutional Review Board of the hospital waived the need for written informed consent from the participants. In addition, information in the patient records was anonymized and de-identified prior to analysis.

### 4.4. Statistical Analyses

Data were entered and analyzed using JMP statistical software (version 9.0, SAS Institute Inc., Cary, NC, USA). Demographic data as well as underlying diseases including asthma, allergic rhinitis, and related covariates were compared between the groups with and without drug eruptions using Fisher’s exact test for categorical variables and the Wilcoxon rank sum test for continuous variables. Cox multivariate logistic regression analysis with stepwise variable selection was performed to identify the risk factors associated with Gemifloxacin-induced skin eruptions. Statistical significance was set at *p* < 0.05.

## 5. Conclusions

This is the first study to report that female asthmatic patients have the highest risk of Gemifloxacin-associated skin eruptions in Asia, and a unique delayed onset skin rash should be highlighted as a potential reaction following treatment with Gemifloxacin.

## Figures and Tables

**Table 1:** Demographic data of patients with Gemifloxacin-induced skin eruptions in Kaohsiung Municipal Ta-Tung hospital from 1 January 2012 to 31 May 2016 (*n* = 1358).

	Non-Skin Rash Group (*n* = 1322)		Skin Rash Group (*n* = 36)		Crude Odds Ratio(OR) (95% Confidence Interval (CI))	*p* Value
	*n*	(%)	*n*	(%)		
Age, years						
<40	232	(97.1)	7	(2.9)	Ref.	
≧40	1090	(97.4)	29	(2.6)	0.88 (0.38–2.04)	0.768
Gender						
Male	699	(98.3)	12	(2.6)	Ref.	
Female	623	(96.3)	24	(3.7)	2.24 (1.11–4.53)	0.021 *
Co-morbidities						
Asthma						
No	1062	(97.8)	24	(2.2)	Ref.	
Yes	260	(95.6)	12	(4.4)	2.04 (1.01–4.14)	0.043 *
Allergic rhinitis						
No	1025	(97.6)	25	(2.4)	Ref.	
Yes	297	(96.4)	11	(3.6)	1.52 (0.74–3.12)	0.253
Drugs allergy NSAID allergy						
No	1287	(97.4)	34	(2.6)	Ref.	
Yes	35	(94.6)	2	(5.4)	2.16 (0.50–9.36)	0.302
Allergy to other antibiotics						
No	1262	(97.2)	36	(2.8)	NA	----
Yes	60	(100)	0	(0.0)		
Allergy to other drugs						
No	1249	(97.4)	33	(2.6)	Ref.	
Yes	73	(96.1)	3	(3.9)	1.56 (0.47–5.19)	0.473
Gemifloxacin treatment, days						
≦7	1214	(97.5)	30	(2.5)	Ref.	
>7	108	(94.7)	6	(5.3)	2.25 (0.92–5.52)	0.077

Note: Allergy to other antibiotics indicates patients with a history of allergy to non-fluoroquinolone antibiotics. Allergy to other drugs indicates allergy to drugs other than antibiotics or NSAIDs. NSAID, Non-Steroid Anti-inflammatory Drug. * *p* < 0.05

**Table 2:** A backward selection logistic regression model to estimate the adjusted odds ratios (aORs) of gender and asthma.

Gender	Asthma	Patients without Skin Rash	Patients with Skin Rash	aOR	(95% CI)	*p* Value
Male	Asthma (−)	568 (98.3%)	10 (1.7%)	Ref.		
Male	Asthma (+)	131(98.5%)	2 (1.5%)	0.87	(0.19–4.04)	0.863
Female	Asthma (−)	494(97.2%)	14 (2.8%)	1.60	(0.71–3.74)	0.261
Female	Asthma (+)	129(92.8%)	10 (7.2%)	4.45	(1.81–10.93)	0.001

Note: 95% CI: 95% confidence interval.
